# Indole-3-propionic acid supplementation during in vitro maturation improves in vitro production of porcine embryos

**DOI:** 10.1186/s12917-025-05049-4

**Published:** 2025-11-03

**Authors:** Yuichiro Nakayama, Takeshige Otoi, Namula Zhao, Suong Thi Nguyen, Nanaka Torigoe, Qyingi Lin, Liu Bin, Oky Setyo Widodo, Theerawat Tharasanit, Kaywalee Chatdarong, Maki Hirata, Megumi Nagahara, Fuminori Tanihara

**Affiliations:** 1 Laboratory of Animal Reproduction, Bio-Innovation Research Center, Tokushima University, 2272-2 Ishii, Ishii-cho, Myozai-gun, Tokushima, 779-3233 Japan; 2 Faculty of Veterinary Medicine, Universitas Airlangga, 60115 Surabaya, Indonesia; 3 Faculty of Veterinary Science, Chulalongkorn University, Bangkok, 10330 Thailand; 4 Department of Veterinary Medicine, College of Coastal Agricultural Sciences, Guangdong Ocean University, Guangdong, 524088 China; 5 Joint Graduate School of Veterinary Sciences, Yamaguchi University, Yamaguchi, 7530841 Japan

**Keywords:** Porcine, In vitro maturation, GSH, ROS, Indole-3-propionic acid

## Abstract

**Background:**

Oxidative stress is a critical factor affecting the maturation and developmental competence of porcine oocytes during in vitro culture, reducing the efficiency of blastocyst formation. Antioxidant supplementation in the culture medium has been proposed to mitigate oxidative stress and improve developmental outcomes. This study aimed to evaluate the effects of indole-3-propionic acid (IPA), a potent antioxidant, on porcine oocyte maturation, fertilization, and subsequent blastocyst development under in vitro conditions.

**Methods:**

Porcine cumulus–oocyte complexes (COCs) were matured for 44 h in maturation medium supplemented with IPA at various concentrations (0.5, 1, 5, and 10 µM). As a control, COCs were cultured in maturation medium containing the IPA dilution vehicle (ethanol) without IPA. After maturation culture, in vitro fertilization and embryo culture were performed to evaluate fertilization rate and developmental competence. Meiotic competence, fertilization status, blastocyst formation, and DNA integrity of oocytes and blastocysts were evaluated. To further investigate the protective effect of IPA against oxidative stress, COCs were exposed to 1 mM H_2_O_2_ in a maturation medium supplemented with or without IPA (0.5 µM) during maturation culture. After maturation, intracellular reactive oxygen species (ROS) and glutathione (GSH) levels in oocytes were assessed.

**Results:**

Supplementation with 0.5 μM IPA in the in vitro maturation medium significantly enhanced the rates of oocyte maturation, fertilization, and blastocyst formation compared with the control group (*p* < 0.05). In addition, IPA supplementation at concentrations ranging from 0.5 to 5 µM significantly reduced DNA fragmentation in both matured oocytes and blastocysts. Furthermore, IPA supplementation effectively reduced the intracellular ROS levels elevated by H_2_O_2_ while significantly increasing intracellular GSH levels (*p* < 0.05). These findings indicate that IPA supplementation protects porcine oocytes from oxidative stress during in vitro maturation by reducing ROS generation and enhancing GSH levels. Consequently, IPA improves oocyte quality, fertilization potential, and developmental competence of embryos.

**Conclusions:**

This study demonstrates the potential of IPA as a beneficial supplement to in vitro maturation medium for improving the efficiency of porcine embryo production by enhancing oocyte quality and protecting against oxidative damage.

## Background

In vitro production of porcine oocytes and embryos is an essential tool in reproductive biology and animal breeding. Furthermore, due to their physiological and anatomical similarities to humans, pigs serve as valuable large-animal models for biomedical studies. In recent years, significant progress has also been made in developing pigs as models for xenotransplantation. Therefore, improving the efficiency and quality of the in vitro production of porcine oocytes, their meiotic competence and subsequent embryo development, is of significant importance for both agricultural productivity and advancements in human medicine [1–3].

Porcine embryos cultured in vitro exhibit a higher incidence of apoptosis compared to those developed in vivo, resulting in reduced embryo quality [4]. A significant cause is the oxidative stress induced by excessive reactive oxygen species (ROS), which exert a detrimental effect on oocytes and embryos development [4–6]. Most ROS are generated during the mitochondrial electron transport chain, where incomplete oxygen reduction leads to superoxide formation [7]. Superoxide is converted into hydrogen peroxide (H₂O₂) by superoxide dismutase and further into hydroxyl radicals, which induce lipid peroxidation, DNA damage (particularly double-strand breaks), and inflammatory cytokine activation. When mitochondrial membrane permeability changes, ROS are released into the cytoplasm, leading to widespread cellular damage [8, 9]. Glutathione (GSH) is synthesized from glutamate, cysteine, and glycine via the γ-glutamyl cycle in the cells [10]. GSH suppresses oxidative stress by transferring electrons to ROS (such as H_2_O_2_ and peroxides), reducing toxicity [11]. In vivo, the regulation of ROS is mediated by antioxidant enzymes such as catalase, as well as non-enzymatic antioxidants like GSH, which suppress excessive ROS production [12–14]. Therefore, antioxidants supplementation in the in vitro culture medium is crucial for protecting oocytes and embryos from oxidative stress and enhancing maturation and blastocyst formation rates, as well as reducing apoptosis [15–17].

Indole-3-propionic acid (IPA) is an enteric intestinal microbiome-derived deamination product of tryptophan produced by intestinal bacteria [18, 19]. It exerts intracellular signaling functions, including the decrease in inflammatory cytokines via the aryl hydrocarbon receptor pathway [20, 21] and the protection against gastrointestinal toxicity through the activation of gastrointestinal signaling [18]. Furthermore, the protective effects of IPA on various tissues and cells have been reported [22–25]. In the field of obstetrics, obese women undergoing in vitro fertilization (IVF) exhibit decreased IPA levels in follicular fluid and serum [26]. This report suggests that IPA has a notable antioxidant function, potentially impacting oocytes. However, we found no published studies, in pigs or other species, that have directly examined the effects of IPA supplementation during IVM, IVF, or IVC. Therefore, the potential effects of IPA on the meiotic competence and developmental capacity of oocytes remain unclear.

This study aims to investigate the effects of IPA supplementation in the in vitro maturation (IVM) medium on the meiotic competence, fertilization, blastocyst formation, and DNA integrity of porcine oocytes, as well as on intracellular levels of ROS and GSH.

## Methods

### IVM

The oocyte collection and IVM procedures were conducted as previously described [16], with minor modifications. Briefly, the ovaries collected from pre-pubertal gilts (Landrace × Large White × Duroc breeds) at a local slaughterhouse were immersed in a 0.9% saline solution maintained at 33 °C and transported to the laboratory within 2 h. Cumulus-oocyte complexes (COCs) were collected from follicles (3–6 mm in diameter). Only COCs with uniform dark-pigmented ooplasm and intact cumulus cell mass with at least three layers were selected for the experiment. The maturation culture medium consisted of tissue culture medium 199 with Earle’s salts (TCM 199; Thermo Fisher Scientific, Waltham, MA, USA) as the basal medium, supplemented with 10% (v/v) porcine follicular fluid, 50 µM sodium pyruvate (Sigma-Aldrich, St. Louis, MO, USA), 2 mg/mL D-sorbitol (Fujifilm Wako Pure Chemical Corporation, Osaka, Japan), 10 IU/mL equine chorionic gonadotrophic hormone (eCG; Kyoritsu Seiyaku, Tokyo, Japan), 10 IU/mL human chorionic gonadotropin (hCG; Kyoritsu Seiyaku), and 50 µg/mL gentamicin (Sigma-Aldrich). Approximately 50 COCs were transferred into 500 µL of a culture medium covered with mineral oil in a four-well dish (Thermo Fisher Scientific) and incubated for 22 h at 39 °C with 5% CO₂. Subsequently, the COCs were transferred into a maturation medium without eCG and hCG supplementation, where they were further incubated for 22 h.

### Analysis of oocyte nuclear maturation and DNA fragmentation

**Fig. 1:**
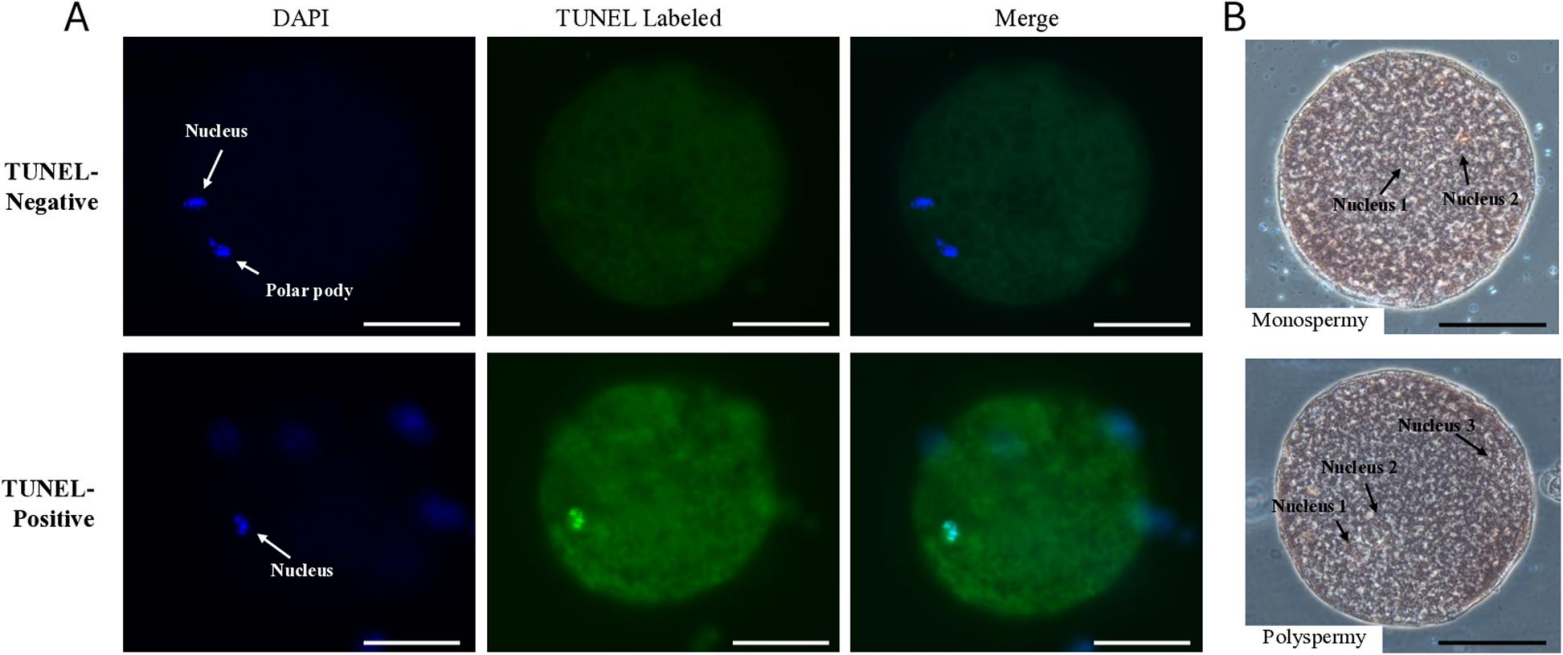
**A** Representative images of DNA-fragmented (TUNEL-positive) and DNA-intact (TUNEL-negative) oocyte after in vitro maturation. **B** Representative images of fertilization assessment after in vitro fertilization. Scale bar indicates 50 µm

Following IVM, terminal deoxynucleotidyl transferase nick-end labeling (TUNEL) staining was performed on a COC subset to assess nuclear maturation and DNA fragmentation, as previously described [27]. Briefly, approximately 20 COCs were denuded of cumulus cells using 150 IU/mL of hyaluronidase and mechanical pipetting. The oocytes were fixed in 3.7% (w/v) paraformaldehyde (PFA) in phosphate buffer saline (PBS) overnight at 4 °C and subsequently permeabilized with 0.1% Triton-X100 for 1 h at 25 °C. Subsequently, they were incubated overnight at 4 °C in PBS containing 10 mg/mL bovine serum albumin. Next, the oocytes were incubated in fluorescein-conjugated 2-deoxyuridine-5-triphosphate (dUTP) and terminal deoxynucleotidyl transferase (TdT) (TUNEL reagent; Roche Diagnostics Corp., Tokyo, Japan) for 1 h at 38 °C. After TUNEL staining, the oocytes were counterstained with 1 µg/mL 4′,6-diamidino-2-phenylindole (DAPI; Thermo Fisher Scientific) for 10 min, treated with an anti-bleaching solution (Slow-Fade; Molecular Probes Inc., Eugene, OR, USA), mounted on a glass slide, and sealed with clear nail polish. For the negative controls, samples were incubated with fluorescein-dUTP without the addition of TdT. The labeled oocytes were examined using an epifluorescence microscope (Eclipse 80i; Nikon, Tokyo, Japan) equipped with epifluorescence illumination. The oocytes were determined to be in the germinal vesicle, condensed chromatin, metaphase I, anaphase I–telophase I, or metaphase II (MII) stage according to the chromatin configuration based on DAPI staining. The oocytes that reach the MII stage were considered mature. To evaluate DNA damage in all oocytes after IVM, the TUNEL-labeled nuclei were visualized, and DNA fragmented nuclei were directly counted from microscopic images (Fig. 1A). The proportion of oocyte containing fragmented nuclei was calculated relative to the total number of oocytes and compared among groups.

### Evaluation of ROS and GSH in oocyte cytoplasm after maturation culture

ROS and GSH levels in oocytes after IVM were determined using the protocol described by Jeon et al. [28]. Briefly, COCs were collected after 44 h of IVM, denuded of cumulus cells using hyaluronidase, and incubated in an IVM medium containing 10 µM 2′,7′-dichlorodihydrofluorescein diacetate (H2DCFDA, D399; Thermo Fisher Scientific) for 15 min to assess ROS level or 10 µM 4-chloromethyl-6,8-difluoro-7-hydroxycoumarin (CMF2HC, C12881; Thermo Fisher Scientific) for 30 min to assess GSH level. Incubations were conducted at 38.5 °C in 5% CO₂, in the dark. After incubation, the oocytes were washed with 0.1% (w/v) polyvinyl alcohol in PBS and placed in 10 μL microdrops. Fluorescence signals were detected using a fluorescence microscope equipped with epifluorescence illumination, with green (465–495 nm as the excitation wavelength and 515–555 nm as the emission wavelength) and blue (340–380 nm as the excitation wavelength and 435–485 nm as the emission wavelength) fluorescence signals for ROS and GSH, respectively. Images were saved as graphic files in the TIFF format. Fluorescence pixel intensity was analyzed using ImageJ software (version 1.54g; National Institutes of Health, Bethesda, MD, USA) as previously described [29, 30]. A region of interest (ROI) was defined for each target image, and the mean fluorescence intensity within the ROI was calculated. These mean values were then used for statistical comparisons between the control and treatment groups. For all measurements, the entire oocyte cytoplasm was designated as the ROI to quantify pixel intensity.

### IVF and fertilization assessment

After IVM, the COCs were washed with a porcine fertilization medium (PFM; Functional Peptide Institute, Yamagata, Japan), and approximately 50 COCs per well were transferred into each four-well dish (filled with 500 µL of PFM containing frozen-thawed spermatozoa at a final concentration of 1 × 10^6^ sperm/mL per well and covered with mineral oil) and co-cultured for 5 h at 39 °C in a humidified incubator with 5% CO₂, 5% O₂, and 90% N₂. To evaluate the fertilization status 10 h post-initial IVF, putative zygotes were denuded of cumulus cells via mechanical pipetting, fixed, and subjected to staining with orcein acetate (1% orcein in 45% acetic acid). After fixation, the putative zygotes were examined under microscopic scrutiny to evaluate the fertilization. The zygotes containing female and male pronuclei were considered to be fertilized and were classified as monospermic or polyspermic fertilized based on the number of sperm heads or pronuclei in the cytoplasm [16] (Fig. 1B).

### In vitro culture and assessment of resulting blastocysts

Following IVF, adherent spermatozoa and cumulus cells were removed from putative zygotes via pipetting. Subsequently, the putative zygotes were incubated with 500 µL of porcine zygote medium-5 (Functional Peptide Institute) at 39 °C, under 5% CO₂, 5% O₂, and 90% N_2_ atmospheric conditions for 72 h. Only cleaved embryos were selected 72 h after IVF and transferred into a porcine blastocyst medium (Functional Peptide Institute) and further cultured for 4 days to assess the blastocyst formation rate. Next, the developed blastocysts were subjected to TUNEL assay, as described above. The DNA fragmentation index was calculated by dividing the number of cells containing DNA-fragmented nuclei (labeled with TUNEL) by the total number of cells.

### Experimental design

#### Experiment 1

To evaluate the effects of IPA supplementation on the in vitro maturation, fertilization, and development of porcine oocytes, COCs were cultured in an IVM medium supplemented with 0.5, 1, 5, or 10 µM IPA (57,400–56-F; Sigma-Aldrich), dissolved in ethanol. The final concentration of ethanol in the IVM medium was 0.1%. The concentration of IPA was determined with reference to the study by Ruebel et al. (2019) [26]. While its physiological concentration in follicular fluid in vivo is typically in the nanomolar range, we increased it to the micromolar range in the in vitro culture system. This adjustment accounted for potential losses from degradation, binding, and dilution, and was intended to facilitate detection of biologically relevant activity. As a control, COCs were cultured in maturation medium containing the 0.1% IPA dilution vehicle (ethanol) without IPA. After maturation culture for 44 h, the COCs were fertilized and cultured continuously. Meiotic competence, fertilization status, blastocyst formation, and DNA integrity of both oocytes and blastocysts were evaluated. All experiments were repeated more than four times. In total, 2,580 oocytes were used in Experiment 1, with more than 500 oocytes analyzed per group. Since IVM, IVF, and IVC are performed sequentially, approximately 100 COCs were used per replicate, allowing for the assessment of IVM, IVF, and IVC, which required about 20, 20, and 60 oocytes, respectively.

#### Experiment 2

To assess the protective effect of IPA on H_2_O_2_-induced oxidative stress in porcine oocytes, the COCs were exposed to 1 mM H_2_O_2_ (Fujifilm Wako Pure Chemical Corporation) [31] in a maturation medium supplemented with or without IPA (0.5 µM) during IVM. The concentration of 0.5 µM IPA was selected, as Experiment 1 demonstrated significant improvements compared with the control group in all evaluated parameters, including maturation, fertilization, blastocyst formation, and DNA fragmentation rates. After IVM, the ROS and GSH levels of oocytes were assessed, as described above. All experiments were repeated more than four times. In Experiment 2, a total of 911 oocytes were used, with more than 200 analyzed per group. Assessments of maturation rate, ROS, and GSH levels required approximately 30, 15, and 15 oocytes, respectively, and were performed using ~ 60 COCs per replicate.

### Statistical analysis

Statistical significance was inferred using analysis of variance, followed by Fisher’s exact least significant difference test using STATVIEW (Abacus Concepts, Inc., Berkeley, CA, USA). The percentage data were subjected to arcsine transformation before statistical analysis. Data were expressed as the mean ± standard error of the mean. Differences with a *p-*value of < 0.05 were considered significant.

## Results

### Effect of IPA supplementation on oocyte maturation, fertilization and DNA fragmentation after IVM

The maturation rates of porcine oocytes after IVM, and fertilization rates after IVF were significantly increased in the group supplemented with 0.5–5 µM IPA compared with that in the control group (*p* < 0.05, Table 1). There were no significant differences in monospermic fertilization rates among the groups, regardless of IPA concentration. Furthermore, the DNA fragmentation rates after IVM were significantly decreased regardless of the IPA concentration supplemented into the IVM medium compared with that in the control group (*p* < 0.05).

**Table 1:** Effects of indole-3-propionic acid (IPA) supplementation during in vitro maturation on the maturation, DNA fragmentation, and fertilization in porcine oocytes*

Concentration of IPA (µM)**	In vitro maturation			In vitro fertilization		
	No. of examined oocytes	No. (%) of oocytes with MII***	No. (%) of oocytes with DNA-fragmented nucleus	No. of examined oocytes	No. (%) of oocytes	
					Total fertilization	Monospermy****
Control	105	55 (51.9 ± 1.7)^a^	14 (13.9 ± 1.9)^a^	103	38 (37.1 ± 2.9)^a^	22 (57.9 ± 3.2)
0.5	113	79 (69.4 ± 2.2)^b^	7 (6.0 ± 1.5)^b^	114	65 (57.0 ± 1.7)^b^	34 (52.2 ± 2.6)
1	116	80 (68.6 ± 2.7)^b,c^	8 (6.8 ± 1.6)^b^	116	68 (58.5 ± 2.3)^b^	34 (50.4 ± 2.6)
5	111	69 (61.9 ± 2.8)^c,d^	6 (5.2 ± 1.2)^b^	113	55 (49.0 ± 1.8)^c^	31 (57.1 ± 5.2)
10	101	58 (57.5 ± 3.3)^a,d^	3 (2.7 ± 1.2)^b^	107	47 (43.8 ± 3.6)^a,c^	27 (57.5 ± 2.1)

^*^All experiments were repeated more than four times. Data are expressed as the mean ± standard error of the mean (SEM)

^**^As a control, cumulus-oocyte complexes were cultured in maturation medium containing the IPA dilution vehicle (ethanol) without IPA

^***^MII, metaphase II

^****^The proportion of monospermic fertilization was calculated by dividing the number of monospermic fertilized oocytes by the number of fertilized oocytes

^a–d^The values with different superscript letters in the same column are significantly different (*p* < 0.05)

### Effects of IPA supplementation on embryonic development and DNA fragmentation in resulting blastocysts

**Fig. 2:**
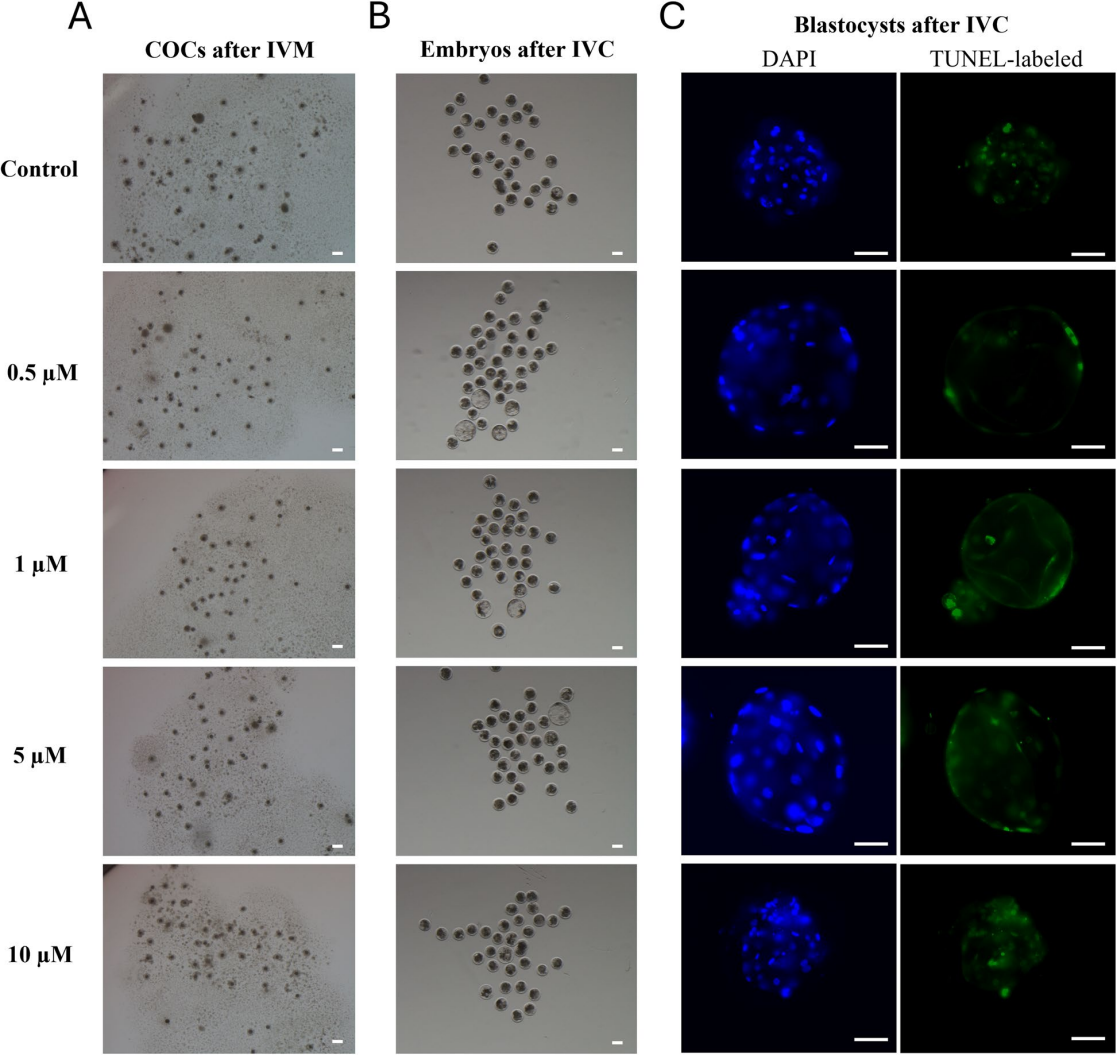
Representative images of oocytes and embryos in Experiment 1. **A** Morphological images of cumulus oocyte complexes (COCs) after in vitro maturation (IVM) in each experimental group. **B** Morphological images of cleaved embryos and blastocysts after in vitro culture (IVC) in each experimental group. **C** Representative DAPI and TUNEL staining images of blastocysts in each experimental group. Scale bar indicates 100 µm

The blastocyst formation rate was significantly increased in the 0.5 µM IPA group compared with that in the control group (*p* < 0.05, Table 2 and Fig. 2). Moreover, the DNA fragmentation index of blastocysts was significantly decreased in the 0.5–5 µM IPA groups compared with that in the control group (*p* < 0.05). There were no significant differences in the average cell number of blastocysts.

**Table 2:** Effect of indole-3-propionic acid (IPA) supplementation during in vitro maturation on the development and DNA fragmentation in porcine embryos*

Concentration of IPA (µM)**	No. of examined oocytes	No. (%) of oocytes developed to blastocysts	Total cell number of blastocysts	DNA fragmentation index (%)***
Control	317	7 (2.3 ± 0.8)^a^	50.6 ± 9.9	7.9 ± 3.0^a^
0.5	297	29 (9.8 ± 2.6)^b^	44.3 ± 4.0	3.7 ± 0.7^b,c^
1	278	15 (5.8 ± 2.7)^a,b^	36.6 ± 4.1	1.9 ± 0.7^c^
5	294	16 (5.6 ± 1.1)^a,b^	42.4 ± 3.9	3.3 ± 1.0^b,c^
10	295	17 (5.8 ± 1.0)^a,b^	37.2 ± 3.4	6.1 ± 1.4^a,b^

^*^All experiments were repeated more than four times. Data are expressed as the mean ± standard error of the mean (SEM)

^**^As a control, oocytes were cultured in maturation medium without IPA

^***^The DNA fragmentation index was defined as the ratio of the number of cells containing DNA-fragmented nucleus and the total number of cells in a blastocyst

^a–^^c^Values with different superscript letters in the same column are significantly different (*p* < 0.05)

### Protective effect of IPA against H₂O₂-induced oxidative stress during IVM

**Fig. 3:**
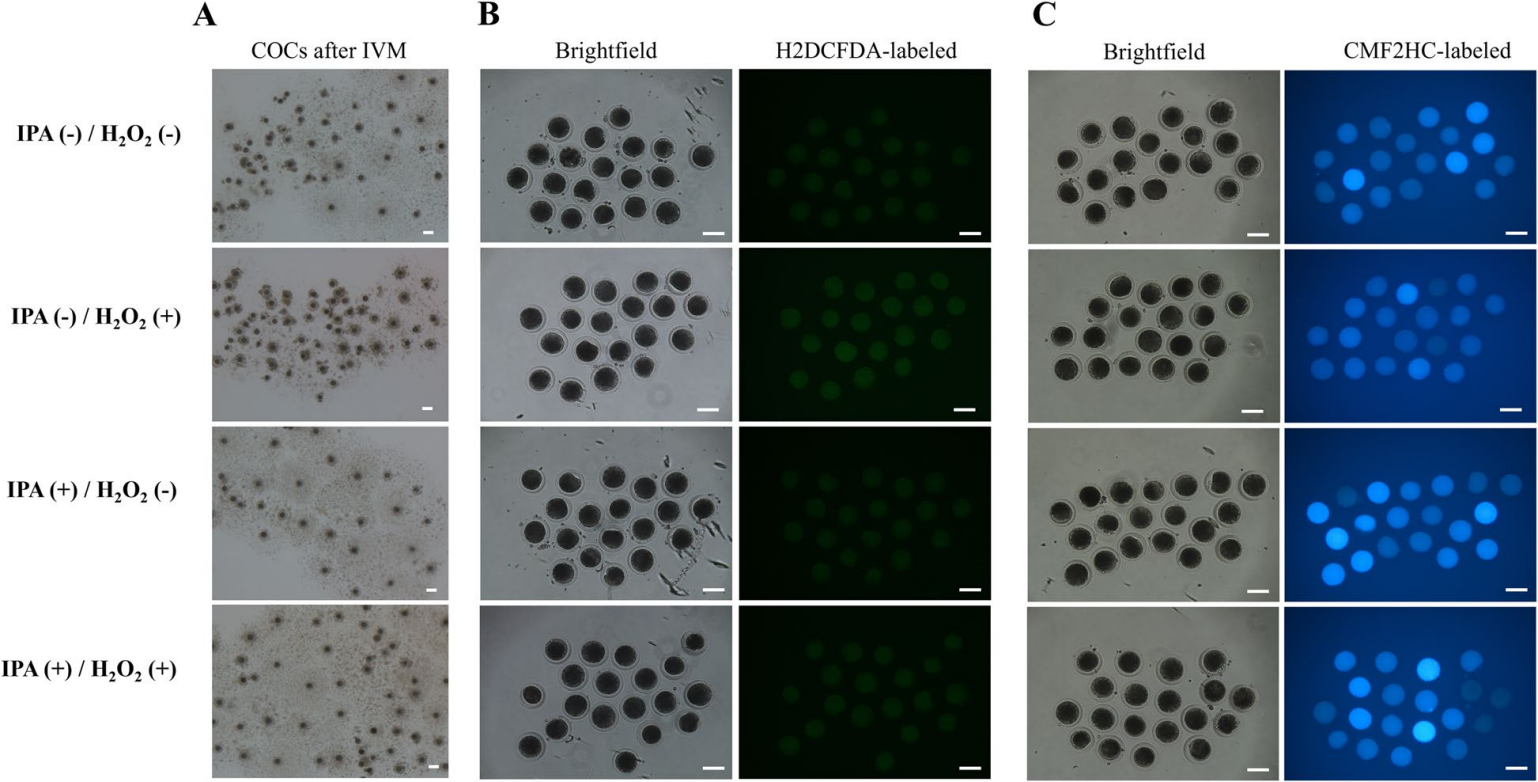
Representative images in each group after in vitro maturation (IVM) supplemented with Indole-3-propionic acid (IPA) in Experiment 2. **A** cumulus oocyte complexes. **B** reactive oxygen species (ROS) staining. **C** glutathione (GSH) staining. Scale bar indicates 100 μm

In the second experiment, the maturation rate of the oocytes cultured in the IVM medium containing H_2_O_2_ and IPA was significantly higher than that in the group cultured in the IVM medium containing only H_2_O_2_ (*p* < 0.05, Table 3 and Fig. 3). Furthermore, IPA supplementation in the IVM medium containing H₂O₂ significantly reduced intracellular ROS levels and increased GSH levels compared with those in the group without IPA supplementation (*p* < 0.05).

**Table 3:** Effect of indole-3-propionic acid (IPA) and H₂O₂ supplementation during in vitro maturation on the maturation rate, intracellular reactive oxygen species (ROS) level, and intracellular glutathione (GSH) level in porcine oocytes*

Supplementation of		No. of examined oocytes*	No. (%) of oocytes with MII***	ROS		GSH	
IPA	H_2_O_2_			No. of examined oocytes	Pixel intensity (A.U.)**	No. of examined oocytes	Pixel intensity (A.U.)**
—	—	114	55 (46.9 ± 3.3)^a^	56	10.6 ± 0.2^a^	58	85.8 ± 1.1^a^
—	+	115	10 (8.4 ± 2.4)^b^	56	12.9 ± 0.1^b^	59	87.8 ± 1.1^a^
+	—	110	73 (66.0 ± 2.7)^c^	53	8.3 ± 0.1^c^	56	92.0 ± 0.9^b^
+	+	117	59 (50.0 ± 1.3)^a^	57	8.8 ± 0.1^d^	60	96.3 ± 0.9^c^

^*^All experiments were repeated more than four times. Data are expressed as the mean ± standard error of the mean (SEM)

^**^Fluorescence intensities of images were measured to quantify pixel intensity (A.U.) using NIH ImageJ software

^a–^^d^Values with different superscript letters in the same column are significantly different (*p* < 0.05)

## Discussion

**Fig. 4:**
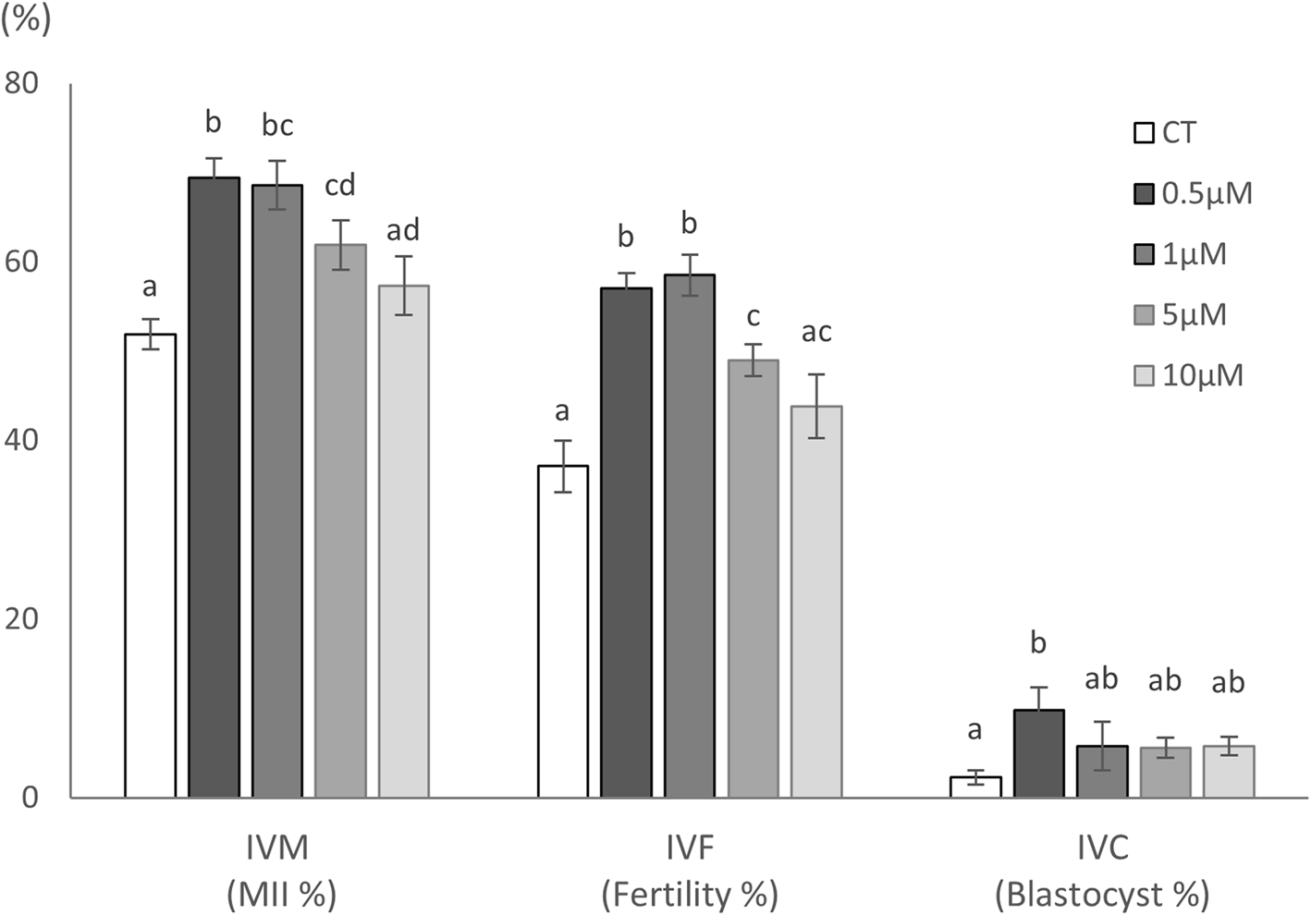
Summary of the effects of indole-3-propionic acid (IPA) supplementation across different stages of porcine in vitro embryo production. a−d Values with different superscript letters in the same column were significantly different (*p* < 0.05). Means ± SEM are presented of all data

We investigated the effect of IPA supplementation in the IVM medium on meiotic competence and embryonic development of porcine oocytes. Supplementation with 0.5 µM IPA during IVM improved the rates of oocyte maturation, fertilization, and blastocyst formation by suppressing DNA fragmentation (Fig. 4). IPA prevented the decrease in microsomal membrane fluidity in rats caused by free radicals induced by the presence of the carcinogens chromium (III) and hydrogen peroxide [32]. Furthermore, a previous study reported that IPA acts as a chemical chaperone, inhibits endoplasmic reticulum stress, and protects against neuronal cell death [25]. IPA suppresses inflammatory genes (TNF-α, IL-6, iNOS, COX-2) and matrix degrading enzymes (MMP-3, MMP-13, ADAMTS-5) while upregulating cartilage matrix proteins (aggrecan, collagen II), indicating its protective effects via NF-κB pathway inhibition and highlighting the value of qPCR analysis for oxidative stress and apoptosis related markers to further clarify its molecular mechanisms [20]. Our results indicated that IPA prevents oxidative stress in oocytes, contributing to improved oocyte maturation and embryo development in in vitro production.

In this study, we supplemented IPA into the IVM medium. Previous studies in humans have shown that follicular fluid contains IPA, suggesting the possibility that pFF may also contain endogenous IPA and could have influenced the results. However, because the purpose of this study was to compare outcomes among IPA supplemented groups, the same batch of pFF was consistently used across all experimental groups within each experimental day. Therefore, even if endogenous IPA was present, its concentration would have been identical among groups, and we believe it would not have affected the comparative outcomes. In addition, the beneficial effects of IPA may be stage-specific, acting primarily during meiotic maturation, with limited direct influence on cell division or differentiation during later embryonic development [1, 20]. From the IVF results, a significant improvement in the fertilization rate was observed with IPA supplementation; however, there was no significant effect on the monospermy rate. Although IPA may contribute to fertilization, it Does not appear to play a role in preventing polyspermy. Based on these findings, we considered that oocytes are particularly susceptible to oxidative stress during IVM and therefore focused our investigation on this stage. Accordingly, in Experiment 2, we specially examined the effects of IPA during IVM. Nevertheless, it remains important to evaluate the potential effects during IVF and IVC, and this requires future investigation.

Our results showed that the IPA supplementation during the IVM improved the subsequent blastocyst formation rate. However, no significant difference was observed in the average cell number of blastocysts. IPA was supplemented only in the IVM medium, suggesting that its effects were primarily exerted during the early stages of embryonic development, from oocyte maturation to fertilization and early cleavage. IPA may enhance blastocyst formation by protecting oocytes from oxidative damage during IVM. However, this effect does not necessarily translate into an increased cell number, because its antioxidant action primarily improves early developmental competence rather than directly promoting cell proliferation at the blastocyst stage [1, 20]. Furthermore, the significant reduction in DNA fragmentation rates observed in blastocysts following IPA supplementation indicates that IPA may have potential to improve blastocyst quality. However, despite the reduced DNA fragmentation rate observed in the 10 µM group after IVM, blastocyst formation was not improved. These results suggest that excessive amounts of IPA supplementation may adversely affect oocyte maturation and thereby fail to improve blastocyst developmental competence. Further studies are required to evaluate the effect of IPA supplementation in the in vitro culture medium to enhance blastocyst quality. Additionally, in this study, the blastocyst rate was notably low. As the primary aim was to evaluate the antioxidant effect of IPA during IVM, commonly used antioxidants were intentionally excluded. Given the well-documented sensitivity of porcine oocytes to oxidative stress, the absence of antioxidant supplementation likely contributed to the reduced embryonic development observed, which is consistent with our previous findings [27]. Despite the suboptimal conditions, IPA supplementation produced significant improvements in developmental outcomes, underscoring its antioxidant efficacy. Accordingly, we consider that this study offers novel insights into the antioxidant role of IPA in oocyte physiology.

Moreover, Experiment 2 demonstrated that the IPA supplementation in the maturation medium increased GSH production, effectively suppressing the H_2_O_2_-induced rapid increase in ROS. GSH protects oocytes from DNA damage caused by oxidative stress and facilitates normal embryonic development [15, 33, 34]. IPA is highly potent in scavenging hydroxyl radicals in vitro and inhibiting lipid peroxidation, and has been reported to outperform melatonin under certain conditions [24, 35]. We hypothesize that IPA modulates intracellular GSH and ROS levels by activating antioxidant defense mechanisms. Previous studies have demonstrated that IPA can activate the Nrf2 signaling pathway, leading to enhanced expression of antioxidant enzymes and improved cellular redox balance [36, 37]. Therefore, it is plausible that the Nrf2 pathway contributes to the effects of IPA observed in our experiments. However, because the Nrf2 pathway was not directly assessed in this study, further investigations are required to confirm its involvement in oocytes. Our findings are consistent with those of previous studies, which demonstrated the protective effects of antioxidants, such as betulinic acid [38], oleanolic acid [39], limonin, and nobiletin [40], on porcine oocytes, as they reduce ROS and increase GSH in oocytes after IVM, as well as improve their maturation and developmental abilities. However, this study did not include comparative analyses with other antioxidants, including resveratrol and curcumin; therefore, further direct comparisons are required to substantiate claims of superiority for supplementation during in vitro production of embryos. Moreover, another limitation of this study is that the assessment of oxidative stress was restricted to ROS and GSH measurements, without characterization of the underlying genes or signaling pathways. Future studies should include qPCR analyses of oxidative stress- and apoptosis-related genes to clarify the molecular mechanisms by which IPA exerts its effects.

IPA, a metabolite produced by gut bacteria from tryptophan, is recognized as a potent hydroxyl radical scavenger [25, 41]. Therefore, IPA prevents endoplasmic reticulum stress and thereby protects against neuronal cell death [25]. Furthermore, it can protect against the oxidative damage to membrane lipids induced by high iron concentrations in porcine skin [22]. Our study indicated that IPA is a promising substance with effective antioxidant activity in in vitro cultures of porcine oocytes. In a previous study, a decline in serum IPA was observed to be associated with a decrease in follicular fluid [26]; therefore, improving the gut environment for IPA production may improve the oocyte quality in vivo. For pig farming, efficient sow selection with high productivity performance is of economic importance. Biomarkers, such as anti-Müllerian hormone, insulin-like growth factor, and ovarian follicles, are important indicators of the productive performance of sows [42–46]. Our findings suggest that serum IPA levels could serve as an indicator for sow selection. However, this study did not investigate the direct relationship between IPA and sow productivity; therefore, further research is required to elucidate this relationship.

## Conclusions

IPA supplementation in IVM medium improves oocyte quality, fertilization potential, and developmental competence of embryos while mitigating DNA damage in pigs. IPA has the potential to serve as a beneficial supplement to IVM medium for improving the efficiency of porcine embryo production.

## Data Availability

The datasets supporting the conclusions of this article are included within the article.

## Abbreviations

COCs: Cumulus-oocyte complexes
eCG: Equine chorionic gonadotrophic hormone
GSH: Glutathione
hCG: Human chorionic gonadotropin
IPA: Indole-3-propionic acid
IVF: In vitro fertilization
IVM: In vitro maturation
MII: Metaphase II
PFM: Porcine fertilization medium
ROS: Reactive oxygen species
TUNEL: Terminal deoxynucleotidyl transferase nick-end labeling
